# Valorization of a By-Product from the Production of Mechanically Deboned Chicken Meat for Preparation of Gelatins

**DOI:** 10.3390/molecules26020349

**Published:** 2021-01-12

**Authors:** Pavel Mokrejš, Robert Gál, Jana Pavlačková, Dagmar Janáčová

**Affiliations:** 1 Department of Polymer Engineering, Faculty of Technology, Tomas Bata University in Zlín, Vavrečkova 275, 760 01 Zlín, Czech Republic; 2 Department of Food Technology, Faculty of Technology, Tomas Bata University in Zlín, Vavrečkova 275, 760 01 Zlín, Czech Republic; gal@utb.cz; 3 Department of Lipids, Detergents and Cosmetics Technology, Faculty of Technology, Tomas Bata University in Zlín, Vavrečkova 275, 760 01 Zlín, Czech Republic; pavlackova@utb.cz; 4 Department of Processing Control and Applied Computer Science, Faculty of Applied Informatics, Tomas Bata University in Zlín, Nad Stráněmi 4511, 760 05 Zlín, Czech Republic; janacova@utb.cz

**Keywords:** biotechnology, circular economy, extraction, food by-products, food sustainability, gelatin, mechanically deboned chicken meat (MDCM) by-product, sustainability, valorization

## Abstract

In recent decades, food waste management has become a key priority of industrial and food companies, state authorities and consumers as well. The paper describes the biotechnological processing of mechanically deboned chicken meat (MDCM) by-product, rich in collagen, into gelatins. A factorial design at two levels was used to study three selected process conditions (enzyme conditioning time, gelatin extraction temperature and gelatin extraction time). The efficiency of the technological process of valorization of MDCM by-product into gelatins was evaluated by % conversion of the by-product into gelatins and some qualitative parameters of gelatins (gel strength, viscosity and ash content). Under optimal processing conditions (48–72 h of enzyme conditioning time, 73–78 °C gelatin extraction temperature and 100–150 min gelatin extraction time), MDCM by-product can be processed with 30–32% efficiency into gelatins with a gel strength of 140 Bloom, a viscosity of 2.5 mPa.s and an ash content of 5.0% (which can be reduced by deionization using ion-exchange resins). MDCM is a promising food by-product for valorization into gelatins, which have potential applications in food-, pharmaceutical- and cosmetic fields. The presented technology contributes not only to food sustainability but also to the model of a circular economy.

## 1. Introduction

The food industry produces a large amount of waste, which is divided according to its origin into animal and vegetable by-products; depending on processability, it is divided into primary and secondary by-products. The primary animal by-products include mainly parts of the bodies from the slaughter of cattle and pigs, poultry and processing of fish and shellfish. In the slaughter of animals, the waste represents approximately 35% of the animal’s live weight [1]. Secondary animal by-products originate in various processing sectors of the food industry, e.g., in the production of edible collagen packaging, in the production of gelatins and glues, in the processing of eggs and milk and also in the production of feed and in tanneries in the processing of hides into leather or in the production of biomedical materials [2]. Edible by-products (such as liver, heart, stomach, kidney, throat or blood) are commonly used in many countries to prepare a variety of dishes. Inedible by-products pose potential risks to human health and the environment, which must be eliminated either by the safe disposal of these products in rendering plants or by using them for other purposes under strict hygienic conditions [3,4]. On the other hand, most of these by-products can be a highly valuable raw material resource. By-products are rich in natural polymers, e.g., chicken heads, stomachs or paws contain 50–75% protein in dry matter (made up of 83–98% collagen); others, e.g., skins contain a high proportion of fats (up to 85%); vegetable by-products can then be an interesting source of raw materials for carbohydrates, proteins or various secondary substances (essential oils, dyes, and tannins) [5]. By suitable processing technologies, unused parts of animal bodies or plants can thus be processed into usable products; their use will also reduce the economic and environmental burden for producers of this waste. The prepared products can then be widely used, for example, in the food industry, pharmacy, cosmetics, the chemical industry or in the production of feed for livestock and pets [6].

Some protein by-products, especially those that contain collagen, are further utilized. The most frequently processed are skins and bones, which are used primarily for the production of gelatins and collagen hydrolysates, which are widely used, especially in the production of food, food supplements or cosmetics [7,8,9]. Keratin-rich by-products are used to prepare keratin hydrolysates, which are used as functional ingredients in cosmetic products. Waste fats and oils, both of vegetable and animal origin, are processed in the chemical industry for the production of soaps and in cosmetic applications they are an important component of creams or balsams [10,11,12]. They are also widely used in the food industry in the production of meat products and margarines [13] and further for the production of biofuels, lubricants and feeds [14].

Gelatin is one of the most important products, which is made from collagen; the raw materials are most often cowhides, pig skins and bones. Gelatin production is a multi-step process involving the following key operations: chopping/crushing, conditioning, hot-water extraction, concentration, extrusion and drying. The key operations in which collagen is converted into gelatin are conditioning and hot-water extraction. During conditioning, the collagen raw material is processed in a suitable environment; in the course of it, chemical denaturation of collagen occurs—the intermolecular bonds stabilizing the quaternary structure of collagen are broken. The washing of the conditioned raw material is followed by hot-water extraction, during which the collagen is thermally denatured—the bonds stabilizing the tertiary structure of collagen are broken. The result is the preparation of a gelatin solution (polypeptide chains). In practice, the conditioning of the raw material is most often carried out using acids or bases; the enzymatic method of processing is used minimally in industrial practice, although it offers many advantages (e.g., reduction of production costs and lower consumption of chemicals and water) [15,16]. In 2018, the global market consumed 583.4 ktons of gelatin with a total turnover of 2824 million USD. Compared to 2012, in 2018 the consumption of gelatins was about 25% higher; an increase is expected in the coming years, and for 2024, it is expected to increase by up to 40%. From these data, it is clear that beef and pork tissues will not be enough in the future to meet the ever-increasing demand for these raw materials for gelatin production and that it will be necessary to look for suitable alternative collagen raw materials. These include, for example, fish skin and bones or poultry tissues; at present, however, these raw materials represent only 3% of the raw materials from which gelatins are made [17].

In 2018, world poultry production was approximately 127.3 million tons, of which 87.4% was chicken; between 2008 and 2018, the number of slaughtered chickens increased by 28.4% [18]. As poultry meat production increases, so does the amount of by-products produced. The category of previously mentioned edible by-products also includes poultry paws or heads, which, however, are not processed for culinary purposes in the territory of the European Union and North America, as they are not accepted by consumers in these territories for cultural reasons. Nevertheless, studies are known to describe the processing of these parts of the body, whether from chickens, turkeys or ducks into collagen products (gelatins and hydrolysates), which can be used, for example, for food applications [19,20,21,22].

Mechanically deboned meat is a product obtained by removing meat residues from bones or skeletons that remain after deboning the main meat parts from these tissues. Mechanically deboned poultry meat is most often obtained; the raw materials are poultry carcasses, necks, wings, and offal but also skins or whole chickens [23]. Mechanically deboned meat residue contains proteins, minerals and fat and is therefore most often processed as an ingredient in the production of feed for livestock. As with other unused poultry by-products, there is an effort to use this residue to produce products with the highest possible added value, which can represent an interesting economic benefit for by-product producers. Studies are known to describe the preparation of gelatins from mechanically deboned chicken meat (MDCM) by-product. The conditioning of the starting material takes place in an alkaline medium or a combination of alkaline and acidic media; yields of extracted gelatins range from 6 to 16% depending on the extraction conditions [24,25,26].

It is clear that the by-products of the food industry represent a hidden raw material potential of nutrients—fats, proteins and other bioactive substances—which can be further used, e.g., in food industry or cosmetics. Many tissues contain a high proportion of proteins, especially collagen, and are therefore suitable for the production of gelatins. At the moment, procedures for processing certain poultry tissues (rich in collagen) into gelatins and collagen hydrolysates are already known [20,21,22]. However, the processing of residues from the production of MDCM into gelatins is not currently implemented in industrial practice as there are still a very limited number of studies dealing with this issue; in addition, the procedures for processing the raw material are based on the use of acids or bases, which represents a certain burden on the environment. Our research group has been focusing on development of biotechnological processes for valorization of solid poultry by-products (especially chicken paws, heads, stomachs and skins) rich in proteins, particularly collagen, and the subsequent use of prepared collagen products (gelatins and hydrolysates) in food and cosmetic applications [27,28,29,30]. The aim of this manuscript is to verify the possibilities of processing MDCM by-product into gelatins with enzymatic treatment of the starting material, which has not been used for these purposes so far. The specific hypotheses tested are as follows. (a) Gelatins properties will be comparable with gelatins prepared from poultry by-product tissues and from bovine or porcine tissues, either using traditional (acid or base) or alternative processing methods. (b) The yields of gelatins will correspond to the potential of the starting material and will be at least comparable with the yields of gelatins prepared from the same or similar tissues.

## 2. Results and Discussion

The schedule of experiments and results of the processing of mechanically deboned chicken meat (MDCM) by-product into gelatins is given in Table 1. Table 2 shows the results of analysis of variance for gelatin yield, gelatin gel strength, gelatin viscosity and gelatin ash content.

### 2.1. The Influence of Process Variables on the Yield and Properties of Gelatins

Regression equations in uncoded units for the 1st gelatin fraction yield, gelatin gel strength, gelatin viscosity and gelatin ash content are presented in Table 3.

#### 2.1.1. Gelatin Yield

**Figure 1:**
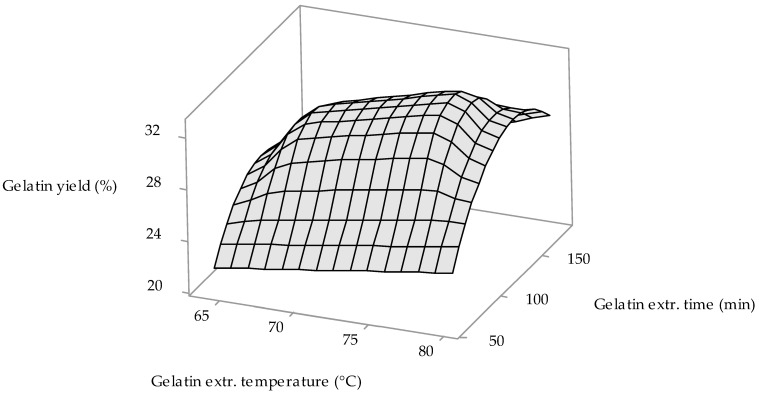
The influence of the gelatin extraction temperature and gelatin extraction time on the 1st gelatin fraction yield.

From factorial regression results (see Table 2) it is obvious that gelatin extraction temperature (Factor B) is a statistically significant factor; *p*-value = 0.044 (confidence level is ≤ 0.05). 3D surface plot in Figure 1 shows the influence of gelatin extraction temperature and gelatin extraction time—factor with *p*-value (0.067) closest to confidence level (0.05)—on the 1st gelatin fraction yield. It is clear from the figure that at the minimum values of both process factors (64 °C and 60 min), the yield of gelatin is minimal (approx. 22%); with an extraction time of up to about 75 min, the yield of gelatin hardly increases with increasing extraction temperature (22–24%). Comparably low yields of gelatins are achieved at extraction temperatures up to 70 °C and long extraction times (above 170 min). Conversely, gelatin yields increase at extraction times in the range of 100–140 min with increasing extraction temperatures; at temperatures of 74–78 °C, the maximum yield of gelatins is achieved (≈32%).

The results of gelatin yields from MDCM by-product are in certain cases lower; on the other hand, in certain cases higher, depending on the processing conditions, in comparison with the yields of gelatins prepared from chicken paws (18–38%) and from chicken heads (20–36%) using analogous biotechnological approach proposed by the authors [27,30]. From the available literature, it is possible to compare the results of gelatin yields from the same starting material treated with different processing methods as well. According to Erge and Zorba, after demineralization of the raw material (24 h with 3% HCl at 10 °C), followed by alkaline conditioning of the demineralized MDCM residue in 2.9–3.4% NaOH for 48 h at room temperature and optimized water extraction conditions (105–183 min at 76–82 °C), 15.3% gelatin yield was achieved [24]. A similar yield of gelatin (16.0%) was recorded by Rammaya et al., who demineralized the starting material under the same conditions, conditioned with an alkaline method (4.0% NaOH, 72 h at room temperature) and extracted with water at pH 4.0 at 80 °C for 2 h [26]. Rafieian et al. used a higher concentration of HCl (6.73%) in the treatment of the MDCM residue; during this process step (24 h at room temperature), they achieved demineralization and at the same time acid conditioning of the raw material. After extraction with water, under optimal conditions (87 °C, 2 h) gelatins were prepared with a yield of 16.9% [25]. Thus, it is clear that the yields of gelatins prepared from MDCM by-product are approximately 2 times higher according to our technology than when using other methods of conditioning the starting material. Confrontation of the results of gelatin yields prepared from other chicken by-products again favors our technology. After conditioning the chicken feet in an acid medium and extracting the gelatins for 90 min at 70 °C, the yield of gelatins was found to be approximately 12.5% [31]. Similar results were obtained by a study examining the effect of alkaline-acid conditioning of the same type of raw material and extraction with water at 55 °C for 24 h—the yield of gelatins was approx. at 15.5% [32]. In the preparation of gelatins from the skins of chicken legs after acid conditioning (3% CH_3_COOH, 24 h) and 5 h extraction at 60 °C, the yield of gelatin was only 14.1% [33], which is more than 2 times less than the yield of our MDCM by-product gelatin.

#### 2.1.2. Gelatin Gel Strength

**Figure 2:**
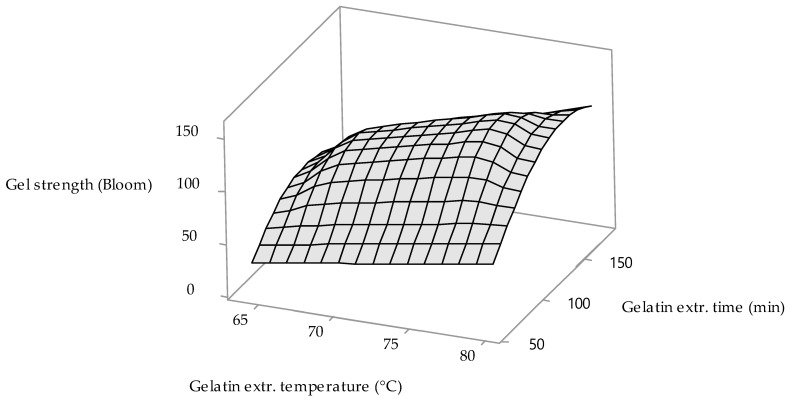
The influence of the gelatin extraction temperature and gelatin extraction time on the gelatin gel strength.

From factorial regression results (see Table 2), it is obvious that all three monitored process variables are statistically significant. Enzyme conditioning time (Factor A) *p*-value equals 0.045, gelatin extraction temperature (Factor B) *p*-value equals 0.029 and gelatin extraction time (Factor C) *p*-value equals 0.044; confidence level for *p*-value ≤ 0.05. 3D surface plot in Figure 2 shows the influence of two statistically most significant factors (gelatin extraction temperature and gelatin extraction time) on the gelatin gel strength. The trend of the influence of the monitored process factors on the strength of gelatin gels is similar to their influence on the yield of gelatins. With short extraction times (up to approximately 80 min), it is clear that gelatins with low gel strength values (20–80 Bloom) are prepared over the entire range of monitored extraction temperatures (64–80 °C). Similarly, at long extraction times (above 160 min) and low extraction temperatures (<69 °C), the strength of gelatin gels ranges from 40 to 80 Bloom. The strength of gelatin gels increases significantly at extraction temperatures above 72 °C, provided that the extraction time is in the range of 100–150 min. At temperatures above 73 °C and an extraction time of 120–140 min, a maximum gelatin gel strength of approximately 140 Bloom is achieved.

A comparison of the strengths of MDCM by-product gelatin gels prepared according to our technology and according to the already mentioned alternative technologies shows that the technological process of raw material processing has a significant effect on the strength of gelatin gels. The highest values of gelatin gel strength (280–1170 Bloom) were achieved according to the procedure of Erge and Zorba, who after demineralization subjected the raw material to alkaline conditioning and extraction of gelatins with hot water [24]. Very high strength values of gelatin gels (320–370 Bloom) were recorded after acid conditioning and extraction of gelatins with hot water [25]. The strength of gelatin gels is significantly higher in gelatins prepared according to the above technological procedures than in our gelatins (approx. 140 Bloom). On the contrary, a comparison with the study of Rammaya et al. [26] favors our technology, because gelatins extracted according to their procedure reach very low values of gel strength (about 62 Bloom). These authors chose parametrically the same procedure for the preparation of gelatins as Erge and Zorba [24], yet the results of the strengths of gelatin gels are significantly lower. These differences can be explained by differences in the process of conditioning the starting material, where Rammaya et al. conditioned the raw material for a longer time in a higher concentration of NaOH solution than Erge and Zorba or compared to our technology, where the starting material was treated with a proteolytic enzyme at pH 6.5–7.0. From the point of view of the structure of collagen after various methods of its conditioning, it can be stated that according to the procedure of Rammaya et al., there was a significant chemical denaturation, including cleavage of the primary structure. Confronting the results of the gelatin gels strengths of our MDCM by-product gelatin and gelatin prepared from chicken feet according to the procedures of Widyasari and Rawdkuen (acid conditioning) [31] and Taufik et al. (alkaline-acid conditioning) [32], it can be stated that the gel strengths of all compared gelatins belong to the category of low–medium Bloom value gelatins: 140 Bloom in MDCM by-product gelatin versus 79–185 Bloom in gelatins prepared according to Widyasari and Rawdkuen and 113–120 Bloom in gelatin prepared according to Taufik et al. Compared to the strength of gelatin gels obtained from the skins of chicken legs [33], our gelatins are of better quality (79 versus 140 Bloom). Significantly higher strength gelatin gels (355 Bloom) were achieved with gelatins prepared from chicken skins, which were however conditioned under mild conditions in an acidic environment (0.15% H_2_SO_4_ and 0.7% CH_3_COOH solutions) and extracted at low temperatures (45 °C) [34].

#### 2.1.3. Gelatin Viscosity

**Figure 3:**
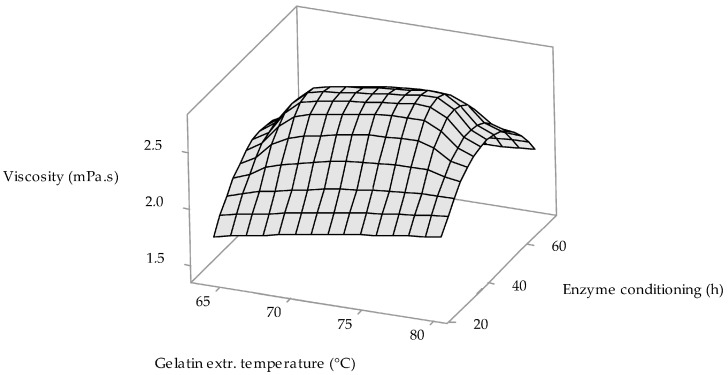
The influence of the gelatin extraction temperature and enzyme conditioning time on the gelatin viscosity.

From factorial regression results (see Table 2), it is obvious that all three monitored process variables have no statistically significant effect on the gelatin viscosity. 3D surface plot in Figure 3 shows the influence of two processing factors, whose *p*-values are closest to assigned confidence level (≤0.05)—gelatin extraction temperature (*p*-value = 0.114) and enzyme conditioning time *p*-value = 0.764)—on the gelatin viscosity. It is clear from the figure that at the minimum and maximum values of the conditioning time (30 and 70 h) the value of the viscosity of gelatins in the whole range of monitored extraction temperatures (64–80 °C) is minimal (1.50–1.75 mPa.s). Note that the prepared gelatins show the highest viscosity (2.50–2.75 mPa.s) with increasing the conditioning time of the raw material to 40 h and its growth up to about 55 h and in a wide range of monitored extraction temperatures (67–78 °C). Thus, it is clear that the gelatin prepared has the same viscosity value over a sufficiently wide range of selected process conditions.

From the available literature on the processing of MDCM residue into gelatins, only Rafieian et al. [25] have performed viscosity testing of prepared gelatins. Gelatins prepared according to their optimized procedure (acid conditioning of the raw material in 6.73% HCl) have a viscosity of 5.9 mPa.s, which is more than 2 times higher value than the gelatins prepared by us according to optimally selected process conditions. Gelatins prepared from chicken feet conditioned by the alkaline-acid method showed an even higher viscosity value (6.3–7.2 mPa.s) [32]. Similar viscosity values (6.5 mPa.s) are also reported by Sompie and Triasih, who prepared gelatins from the skins of acid-conditioned chicken legs [33].

#### 2.1.4. Gelatin Ash Content

**Figure 4:**
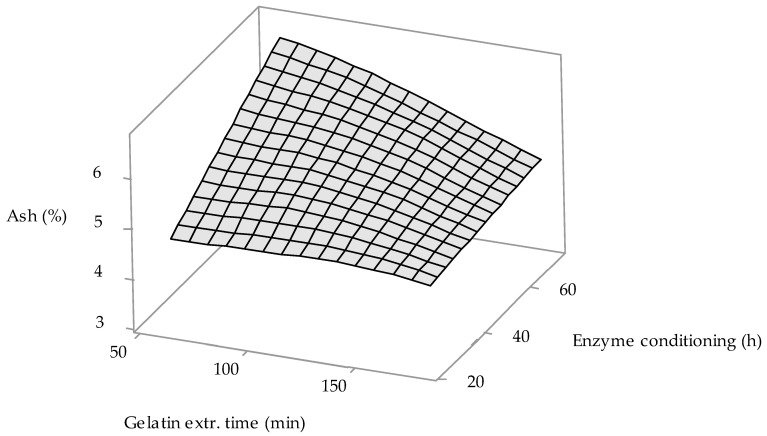
The influence of the gelatin extraction time and enzyme conditioning time on the gelatin ash content.

From factorial regression results (see Table 2), it is obvious that enzyme conditioning time (Factor A) is statistically significant factor, *p*-value = 0.050, which is on the edge of the confidence level (0.05). 3D surface plot in Figure 4 shows the influence of enzyme conditioning time and gelatin extraction time—factor with *p*-value (0.062) closest to confidence level (0.05)—on the gelatin ash content. It is clear from the figure that long conditioning of the raw material (60–70 h) at a short extraction temperature (60–80 °C) will result in a higher ash content in the prepared gelatins (6.0–6.5%). There is a clear trend that as the conditioning time is shortened (to approximately 35 h) and the extraction time is increased (up to approx. 150 min), the ash content decreases to values approaching 5.0%. The lowest ash content in gelatins (4.5–5.0%) is achieved with short conditioning time of raw material (<30 h); the extraction time in this case has no effect on the ash content.

Of the available literature on the processing of MDCM residue into gelatin, ash content values are reported only by Rammaya et al. [26]. Gelatins prepared according to their optimized procedure (demineralization with 3% HCl, alkaline conditioning with 4.0% NaOH and extraction with water at pH 4.0) have a high ash content (39.8%), which is approximately 8 times higher than our MDCM by-product gelatin. Of the other previously cited literature on the preparation of gelatins from chicken by-products, only Sompie and Triasih [33], who used 3% CH_3_COOH to condition the starting material (chicken legskin), report the ash content of gelatins; 9.6% ash content is approximately 2 times higher than our gelatins.

### 2.2. Summarizing the Results and Proposal of Optimal Conditions of Processing of MDCM By-Product into Gelatins

Biotechnological approach in the preparation of gelatins was already published in the manuscripts by the same group of authors [27,35]. The novelty of the present article is related to the optimization of gelatin extraction process with new raw material—mechanically deboned chicken meat (MDCM) by-product. MDCM by-product contains 40% protein in dry matter, of which 80% is collagen. Washing in H_2_O, NaCl, NaOH removes globular proteins, and enzymatic defatting removes the fat fraction; thus, a purified raw material suitable for obtaining collagen or collagen products (gelatin and hydrolysate) is obtained.

Generally, selected technological parameters (within the studied limits) during processing of MDCM by-product into gelatins have an influence on dependent variables. A significant effect of the parameters is evident when evaluating the strength of gelatin gels, where the difference between the minimum and maximum gel strength is approximately 20-fold (158 Bloom versus 8 Bloom). An approximately 2-fold difference was observed when studying the effect of process factors on gelatin viscosity (1.43 mPa.s versus 2.75 mPa.s) and gelatin ash content (3.2% versus 6.7%). Although the smallest (approximately 1.5-fold) difference between the minimum and maximum yield (21.1% versus 32.3%) was recorded when studying the influence of process factors on the gelatin yield, this difference is significant from the point of view of industrial gelatin production, as it brings gelatin manufacturer in an optimally set production process up to 1.5 times higher utilization of the starting material.

From the results of the influence of the monitored process factors on the process efficiency and selected qualitative parameters of gelatins, the optimal process conditions of MDCM by-product processing into gelatins can be summarized as follows. At the maximum values of the monitored process parameters, i.e., 72 h of enzyme conditioning time and extraction of gelatin at 80 °C for 180 min, gelatin with a gel strength of approximately 160 Bloom, with a viscosity of 2.2 mPa.s and an ash content of 4.2% is prepared in a yield of 32%. If we evaluate the results of graphical evaluation of the monitored process factors (Figure 1, Figure 2, Figure 3 and Figure 4) on the yield and quality of prepared gelatins, it can be stated that at enzyme conditioning time within the interval 48–72 h, gelatin extraction temperature within the interval 73–78 °C and gelatin extraction time within the interval 100–150 min, gelatin with approx. 140 Bloom gel strength, viscosity of 2.5 mPa.s and 5.0% of ash content, is prepared. The yield of gelatin (30–32%) corresponds to the potential of the starting raw material. The novelty of the work with respect to what is already published in the biography is that the gelatin yield is approximately 2 times higher than the yields of gelatins prepared from MDCM by-product [24,25,26] or other chicken unused tissues (feet and heads) [19,20,21] by traditional raw material conditioning methods (acidic, alkaline or a combination thereof). Research hypotheses related to gelatin yield have been correctly established and confirmed. The research hypothesis related to the key properties of MDCM by-product gelatin (gel strength and viscosity) cannot be unambiguously confirmed or refuted. The gel strength of our gelatins compared to some gelatins prepared from MDCM by-product or chicken feet and heads is comparable; in this comparison, the research hypothesis was confirmed. On the contrary, compared to gelatins prepared under other process conditions (acidic or alkaline conditioning) from MDCM by-product, the gel strength of our gelatins is lower; in this comparison, the research hypothesis was not confirmed. The viscosity of MDCM by-product gelatin is approximately 2 times lower than that of gelatins prepared from either MDCM by-product or other chicken tissues using other raw material conditioning methods; in this comparison, the research hypothesis was not confirmed.

In terms of gelatin classification, the gelatins prepared by us belong to the category of low-medium gel strength gelatins and to the category of medium viscosity gelatins. Although the ash content of gelatins prepared from MDCM by-product exceeds the limits prescribed for pharmaceutical (2.0%) and food applications (3.0%) [36,37], the ash content of the prepared gelatin solution can be reduced by standard technological operations applied in such cases in gelatin production—deionization using ion-exchange resins [38]. Gelatins with the stated properties are suitable for applications in the food industry, especially in the production of confectionery, such as gums (140–160 Bloom gel strength, 10–15% *w*/*w* gelatin content), wine gums (125–150 Bloom gel strength, 4–8% *w*/*w* gelatin content), meringues (100–150 Bloom gel strength, 2–5% *w*/*w* gelatin content), licorice (100–150 Bloom gel strength, 3–8% *w*/*w* gelatin content), chewy candies (125–150 Bloom gel strength, 0.5–2.5% *w*/*w* gelatin content), toffees (100–150 Bloom gel strength, 0.2–1.0% *w*/*w* gelatin content) or caramels (100–150 Bloom gel strength, 0.2–1.0% *w*/*w* gelatin content) [39,40,41,42]. In the pharmaceutical applications they are suitable for the production of tablets (140–160 Bloom gel strength) as a binding agent [15].

If we compare the yield and properties of gelatins prepared under optimal process conditions with the results of our previous research onto the processing of chicken and hen by-products into gelatins, the following can be stated. The yield of gelatins from MDCM by-product (30–32%) is more or less comparable with the yields of gelatins prepared from chicken paws (18–38%) [27] and from chicken heads (20–36%) [30]; depending on the conditions of their preparation. However, compared to the yield of gelatins prepared from hen paws (8–21%) [35], the yield of gelatins from MDCM by-product is much higher (1.5–4.0 times). The strength of MDCM by-product gelatin gels (140 Bloom) is 1.6–2.3 times lower than gelatins prepared from chicken paws (220–320 Bloom) and 2.0–2.7 times lower than gelatins prepared from hen paws (275–380 Bloom). A large variance of gel strength values (113–355 Bloom) was found in gelatins prepared from chicken heads, depending on the conditions of their preparation, so the result of the comparison cannot be unambiguously stated—1.2 times higher or 2.5 times lower gel strength in favor of MDCM by-product gelatins. The viscosity of MDCM by-product gelatins (2.5 mPa.s) is lower compared to gelatins prepared from chicken paws (3.5–7.5 mPa.s) and to gelatins prepared from hen paws (3.3–7.7 mPa.s); compared to the viscosity of gelatins prepared from chicken heads (1.4–9.5 mPa.s), the gelatin from MDCM by-product is 1.8 times higher on the one hand, but 3.8 times lower on the other hand (depending on the conditions of preparation of chicken heads gelatins). The ash content of MDCM by-product gelatins is about 2.5 times higher than that of gelatins prepared from chicken paws (5.0% versus 2.0%); 1.3–2.2 times higher compared to gelatins prepared from chicken heads (5.0% versus 2.3–3.9%); compared to gelatins prepared from hen paws 2.9 times higher (5.0% versus 1.7%).

The solid residue remaining after gelatin extraction contains minerals and a residual collagen content. By additional hydrolysis, the collagen fraction contained therein can be processed into collagen hydrolysate [43]. Solid residue, with or without residual collagen, is, due to its high mineral content, suitable for applications in compound feed for livestock or as a fertilizer in agriculture.

## 3. Materials and Methods

### 3.1. Materials and Appliances

Mechanically deboned chicken meat (MDCM) by-product was supplied by Raciola, Ltd. (Uherský Brod, Czech Republic). First, by-product material analyses were performed by conventional food methods [44,45,46]. Dry matter content = 38.2 ± 0.7%; in dry matter: protein content 40.3 ± 1.2%, collagen proportion in the protein content 79.9 ± 0.5%, fat content 26.0 ± 1.5% and inorganic solids content 29.6 ± 3.8%. Each analysis was repeated three times; mean values and standard deviations were calculated.

Stevens LFRA Texture Analyzer for measuring gelatin gel strength (Leonard Farnell and Co Ltd., Liverpool, UK), Ubbelohde viscometer (Technisklo Ltd., Držkov, Czech Republic), meat mincer Braher P22/82 (Braher, San Sebastian, Spain), Nedform LT 43 shaker (Nedform, Valašské Meziřící, Czech Republic), Kern 440-47 electronic scale, Kern 770 electronic analytical balance (Kern, Balingen, Germany), analytical mill IKA A 10 labortechnik (IKA, Staufen, Germany), Memmert ULP 400 drying oven (Memmert, Bűchenbach, Germany), Samsung fridge freezer (Samsung, Seoul, South Korea), Whatman no. 1 paper (Sigma Aldrich, Gillingham, UK), WTW pH meter Multical pH 526 (WTW, Weilheim, Germany), heating board Schott Geräte (Mainz, Germany), a metal filter sieve with the size of pores of 1.0 and 2.0 mm (Labor-komplet, Praha, Czech Republic).

Chemicals: NaCl, NaOH, HCl, petroleum ether, ethanol and chloroform (Verkon, Praha, Czech Republic); all chemicals were of analytical grade. Lipolase 100 T^®^, a lipolytic enzyme from Novozymes (Copenhagen, Denmark), was used for defatting MDCM by-product. It is a Thermomyces lanuginosus lipase produced by submerged fermentation of a genetically modified microorganism Aspergillus oryzae with declared activity of 100 KLU/g; optimal activity is in water solutions at temperature 20–45 °C. Protamex^®^, an endoprotease from Novozymes (Copenhagen, Denmark), was used for conditioning of defatted MDCM by-product. It is a Bacillus protease complex with declared activity 1.5 AU/g; optimal working conditions are at pH 5.5–7.5 and temperature ˂60 °C. Enzymes comply with the recommended purity specifications for food-grade enzymes issued by the Joint FAO/WHO Expert Committee on Food Additives (JECFA) and the Food Chemicals Codex (FCC).

### 3.2. Design of Eperiments and Statistical Analysis

The experiments were designed according to factor plans [47]. A full two-level factorial design 2^3^ with one replicate for corner points and two central points (10 experiments) was used to examine the influence of selected process variables during processing of mechanically deboned chicken meat (MDCM) by-product into gelatins. The monitored independent variables were as follows: enzyme conditioning time (factor A), gelatin extraction temperature (factor B) and gelatin extraction time (factor C); levels of variables are presented in Table 1. Studied dependent variables were gelatin yield, gelatin gel strength, gelatin viscosity and gelatin ash content. To compare the results of factor plan, a blind experiment (without enzyme dosage) was performed (experiment No. 11).

Analyses of gelatins were performed in triplicate; mean and standard deviation values were calculated using Microsoft Office Excel 2013 (Microsoft, Denver, CO, USA). To evaluate obtained data, regression analysis was applied to all results using Minitab^®^ 17.2.1 statistical software for Windows (Fujitsu Ltd., Tokyo, Japan). The level of significance was set to 5%, *p*-value ≤ 0.05; factors with a value ≤ 0.05 have an effect on the evaluated process variables with 95% probability. 3D surface plots (Figure 1, Figure 2, Figure 3 and Figure 4) were evaluated by the same software using Akima’s polynomial interpolation method.

### 3.3. Processing of MDCM By-Product into Gelatins

**Scheme 1:**
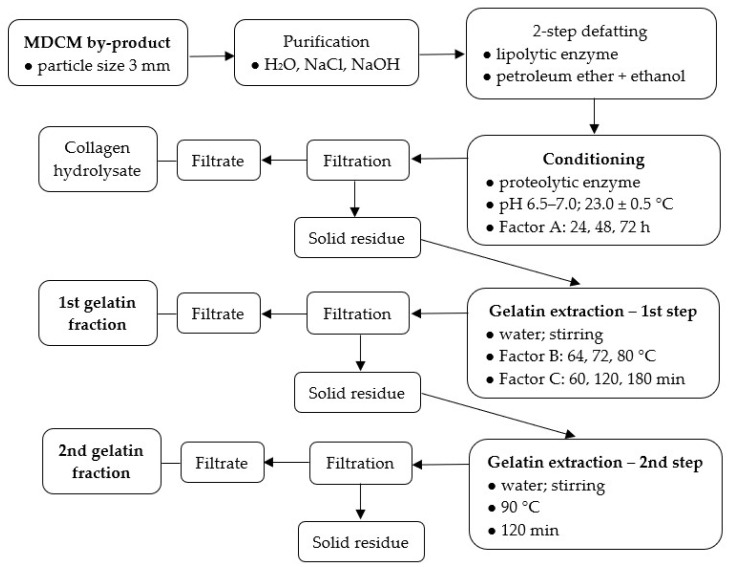
The layout of processing mechanically deboned chicken meat (MDCM) by-product into gelatins.

A general process layout of processing mechanically deboned chicken meat (MDCM) by-product into gelatins is shown in Scheme 1.

Firstly MDCM by-product was purified. Blood residues were removed by washing in running cold water for 3 min. This was followed by the removal of other globular proteins by shaking the raw material mixed in a ratio of 1:6 (*w*/*v*) with 0.2 mol/L NaCl at room temperature for 1.5 h. After washing for 1 min with running cold water, the raw material was mixed in a ratio of 1:6 (*w*/*v*) with 0.03 mol/L NaOH and shaken for 15 h at room temperature, the NaOH solution being changed at 5-hour intervals; glutellins were washed in this way. The raw material was then washed under running cold water for 2 min. This was followed by defatting in two steps. In the first step, the raw material was mixed in a ratio of 1:10 (*w*/*v*) with water, the lipolytic enzyme Lipolase 100 T^®^ was added in a dose of 5.0% (based on the weight of the raw material) and shaken for 48 h at room temperature, changing the water after 12-h intervals and adding a new batch of enzyme. After filtration and washing with cold water for 2 min, the raw material was dried at 35 °C for 24 h. In the second defatting step, the raw material was mixed in a ratio of 1:9 (*w*/*v*) with petroleum ether and ethanol (mixed in a ratio of 1:1, *v*/*v*) and shaken for 20 h at room temperature; after 12 h, the solvent was changed. In the next step, the purified MDCM by-product was subjected to conditioning with a proteolytic enzyme Protamex^®^. The raw material was mixed with water in a ratio of 1:10 (*w*/*v*) and after 20 min of gentle shaking at room temperature, the pH of the mixture was adjusted to 6.5–7.0. Then Protamex^®^ endoprotease was added at a dose of 1.0% (based on the weight of the raw material), and the mixture was shaken gently at 23.0 ± 0.5 °C for a period according to Factor A (24–72 h); during the first 6 h of the enzyme treatment, the pH was checked and, if necessary, adjusted to the prescribed value (6.5–7.0). After filtration (Whatman No. 1 paper), the liquid portion (collagen hydrolysate) was obtained and dried in a thin layer (4 mm) in a circulating air drier at 50.0 ± 0.5 °C. The filtration solid residue was further processed—washed under running water for 2 min, then mixed in a sufficient excess with 0.03 mol/L NaOH and shaken intensively for 10 min, finally washed again under running water for 2 min. During the extraction of the 1st gelatin fraction, conditioned raw material was mixed with water in a ratio of 1:8 (*w*/*v*), the mixture was heated at a rate of dt/dτ = 10 °C/min to the temperature according to Factor B (64, 72, 80 °C), and the gelatin was extracted for a time according to Factor C (60, 120, 180 min). After filtration (filter crucible Simax P16), the solution of the 1st gelatin fraction was immediately heated to a temperature of 95.0 ± 0.5 °C (dt/dτ = 15 °C/min) and maintained at this temperature for 5 min. During the extraction of the 2nd gelatin fraction, the raw material was mixed with water in a ratio of 1:8 (*w*/*v*), the mixture was heated at a rate of dt/dτ = 10 °C/min to the temperature of 90 °C, and the gelatin was extracted for 120 min. After filtration (filter crucible Simax P16), a solution of the 2nd gelatin fraction was obtained. Both gelatin solutions were dried in a thin layer (4 mm) in a circulating air drier at 50.0 ± 0.5 °C; after drying for 48 h, the resulting film was scraped off, weighed and ground to a powder; the prepared gelatins were subjected to further analyzes. The solid residue was dried at 103.0 ± 1.0 °C to constant weight and then weighed.

Total extraction yield (∑Y) and balance error (BE) were calculated according to the following formulas:

(1) $$\sum^{\;}Y={\;Y}_{H}+Y_{G1}+Y_{G2}$$

(2) $$\mathrm{BE}=\;\left|100-\left(Y_{H}+Y_{G1}+Y_{G2}+\mathrm{SR}\right)\right|$$

where Y_H_ is the yield of hydrolysate (%), Y_G1_ is the yield of the 1st gelatin fraction (%), Y_G2_ is the yield of the 2nd gelatin fraction (%) and SR is a solid residue (%). The yield of hydrolysate is calculated as the dry matter content of the prepared hydrolysate relative to the dry matter of MDCM by-product The yields of 1st and 2nd gelatin fractions are calculated as the dry matter content of the prepared gelatins relative to the dry matter of MDCM by-product.

Gelatin gel strength, gelatin viscosity and ash content were determined according to standard procedure for testing of gelatins [48].

## 4. Conclusions

MDCM by-product is a promising by-product for valorization into gelatins. After biotechnological conditioning of the purified raw material in water with the addition of 1.0% (*w*/*w*) endoprotease at pH 6.5–7.0 at room temperature for 48–72 h, the collagen from MDCM by-product is ready for gelatin extraction. Washing the collagen with water is followed by 100–150 min hot-water extraction at 73–78 °C; after filtration and drying, gelatin with 140 Bloom gel strength, 2.5 mPa.s viscosity and 5.0% of ash content is prepared. The yield of gelatins (30–32%) is comparable with the yields of industrially produced gelatins from bovine and porcine tissues and also comparable with the yields of gelatins prepared according to our biotechnological method in the processing of chicken heads and paws. MDCM by-product gelatins belong to the category of low-medium gel strength and viscosity gelatins; such gelatins are made from pork, beef or fish tissues and have irreplaceable use in some food and pharmaceutical applications. MDCM by-product gelatins are particularly suitable for use in the confectionery industry and for the production of pharmaceutical tablets. Moreover, solid residue remained after gelatin extraction may be utilized, e.g., as a feed supplement or fertilizer. A key novelty of the work is that the purified MDCM by-product is treated exclusively with a proteolytic enzyme prior to gelatin extraction, which is an ecology-friendly method; in contrast to competing processes that use acids and/or bases for this purpose. The processing technology of MDCM by-product represents full exploitation of this kind of food by-product (which is not currently optimally utilized) into gelatins and utilizable solid residue; therefore, it meets the concept of circular economy.

## 5. Patents

From the work reported in this manuscript, following patent resulted: Patent CZ 307665-Biotechnology-based production of food gelatine from poultry by-products (2019). At present, the international patent application of the invention under the same name (PCT/CZ2018/050054) is the subject of a research assessment.

## Figures and Tables

**Table 1:** The schedule of experiments and the results of processing of MDCM by-product into gelatins.

	Process Variables			Mass Balance					Gelatin Analysis		
Exp. No.	Factor A (h)	Factor B (°C)	Factor C (min)	Y_H_ (%)	Y_G1_ (%)	Y_G2_ (%)	∑Y (%)	BE (%)	GS (Bloom)	ν (mPa.s)	Ash (%)
1	24	64	60	4.8	21.1	2.1	28.0	3.6	8	1.43	3.23
2	24	64	180	4.2	22.1	2.2	28.5	2.8	28	1.96	4.21
3	24	80	60	4.2	23.7	3.1	31.0	4.1	59	2.11	6.10
4	24	80	180	4.7	24.7	3.7	33.1	3.5	76	1.91	4.90
5	72	64	60	6.2	21.6	2.6	30.4	4.2	42	2.13	6.70
6	72	64	180	6.3	22.8	2.6	31.7	4.8	54	1.43	5.52
7	72	80	60	6.7	24.1	3.6	34.4	2.3	61	1.75	6.30
8	72	80	180	6.8	32.3	6.3	45.4	3.9	158	2.17	4.24
9	48	72	120	5.8	31.7	5.8	43.3	4.5	115	2.66	5.47
10	48	72	120	5.9	31.1	5.2	42.2	4.0	120	2.75	5.30
11 *	48	72	120	1.5	19.1	5.8	26.4	3.0	13	1.54	2.10

Factor A—enzyme conditioning time; factor B—gelatin extraction temperature; factor C—gelatin extraction time; Y_H_—the yield of hydrolysate; Y_G1_—the first gelatin fraction yield; Y_G2_—the second gelatin fraction yield; ∑Y—total extraction yield; BE—balance error; GS—gelatin gel strength; ν—gelatin viscosity; Ash—gelatin ash content; *—a blind experiment (without enzyme dosage).

**Table 2:** Analysis of variance of the experimental design for gelatin yield, gelatin gel strength, gelatin viscosity and gelatin ash content.

	DF	SS	MS	F-Value	*p*-Value
**Gelatin Yield**					
Model	8	175.116	21.8895	121.61	0.070
Factor A (Enzyme conditioning time)	1	10.580	10.5800	58.78	0.083
Factor B (Gelatin extraction temperature)	1	36.980	36.9800	205.44	0.044 ^●^
Factor C (Gelatin extraction time)	1	16.245	16.2450	90.25	0.067
2-way interaction AB	1	5.780	5.7800	32.11	0.111
2-way interaction AC	1	6.845	6.8450	38.03	0.102
2-way interaction BC	1	6.125	6.1250	34.03	0.108
3-way interaction ABC	1	6.125	6.1250	34.03	0.108
Curvature	1	86.436	86.4360	480.20	0.029
Error	1	0.180	0.1800		
Total	9	175.296			
**Gelatin Gel Strength**					
Model	8	19,098.4	2387.30	190.98	0.056
Factor A (Enzyme conditioning time)	1	2592.0	2592.00	207.36	0.045 ^●^
Factor B (Gelatin extraction temperature)	1	6160.5	6160.50	492.84	0.029 ^●^
Factor C (Gelatin extraction time)	1	2664.5	2664.50	213.16	0.044 ^●^
2-way interaction AB	1	72.0	72.00	5.76	0.251
2-way interaction AC	1	648.0	648.00	51.84	0.088
2-way interaction BC	1	840.5	840.55	67.24	0.077
3-way interaction ABC	1	968.0	968.00	77.44	0.072
Curvature	1	5152.9	5152.90	412.23	0.031
Error	1	12.5	12.50		
Total	9	19,110.9			
**Gelatin Viscosity**					
Model	8	1.76495	0.22062	54.47	0.104
Factor A (Enzyme conditioning time)	1	0.00061	0.00061	0.15	0.764
Factor B (Gelatin extraction temperature)	1	0.12251	0.12251	30.25	0.114
Factor C (Gelatin extraction time)	1	0.00031	0.00031	0.08	0.828
2-way interaction AB	1	0.00911	0.00911	2.25	0.374
2-way interaction AC	1	0.04651	0.04651	11.48	0.183
2-way interaction BC	1	0.01901	0.01901	4.69	0.275
3-way interaction ABC	1	0.42781	0.42781	105.63	0.062
Curvature	1	1.13906	1.13906	281.25	0.038
Error	1	0.00405	0.00405		
Total	9	1.76900			
**Gelatin Ash Content**					
Model	8	10.3134	1.28917	89.22	0.082
Factor A (Enzyme conditioning time)	1	2.3328	2.33280	161.44	0.050 ^●^
Factor B (Gelatin extraction temperature)	1	0.4418	0.44180	30.57	0.114
Factor C (Gelatin extraction time)	1	1.4964	1.49645	103.56	0.062
2-way interaction AB	1	3.4322	3.43220	237.52	0.041
2-way interaction AC	1	1.1400	1.14005	78.90	0.071
2-way interaction BC	1	1.1704	1.17045	81.00	0.070
3-way interaction ABC	1	0.2112	0.21125	14.62	0.163
Curvature	1	0.0884	0.08836	6.11	0.245
Error	1	0.0144	0.01445		
Total	9	10.3278			

DF—degree of freedom; SS—sum of squares; MS—mean squares; ^●^—statistically significant factor.

**Table 3:** Regression equations for first gelatin fraction yield, gelatin gel strength, gelatin viscosity and gelatin ash content.

Regression Equations
Y_G1_ (%) = 2.80 + 0.308A + 0.2750B + 0.1242 C − 0.00469AB − 0.004826AC − 0.001823BC + 0.000076ABC + 7.350CtPt
GS (Bloom) = −383.0 + 7.12A + 5.656B + 1.767C − 0.0990AB − 0.06250AC − 0.02448BC + 0.000955ABC + 56.75CtPt
ν (mPa.s) = −7.64 + 0.1928A + 0.1273B + 0.06471C − 0.002585AB –0.001498AC − 0.000862BC + 0.000020ABC + 0.8437CtPt
Ash (%) = −23.21 + 0.4215A + 0.3700B + 0.1115C − 0.005104AB − 0.001278AC − 0.001474BC + 0.000014ABC + 0.2350CtPt

Y_G1_—the first gelatin fraction yield; GS—gelatin gel strength; ν—gelatin viscosity; Ash—gelatin ash content; A—enzyme conditioning time; B—gelatin extraction temperature; C—gelatin extraction time.

## Data Availability

The data presented in this study are available on request from the corresponding author.

## Sample Availability

Samples of the compounds are not available from the authors.
